# The Annexin a2 Promotes Development in Arthritis through Neovascularization by Amplification Hedgehog Pathway

**DOI:** 10.1371/journal.pone.0150363

**Published:** 2016-03-10

**Authors:** Jun Yi, Yan Zhu, Yin Jia, Hongdie Jiang, Xin Zheng, Dejing Liu, Shunxiang Gao, Mingjuan Sun, Bo Hu, Binghua Jiao, Lianghua Wang, Kaihui Wang

**Affiliations:** 1 Department of Medical Genetics, Second Military Medical University, Shanghai, China; 2 Department of Changhai Hospital, Second Military Medical University, Shanghai, China; 3 Department of Biochemistry and Molecular Biology, Second Military Medical University, Shanghai, China; Queen Mary University of London, UNITED KINGDOM

## Abstract

The neovascularization network of pannus formation plays a crucial role in the development of rheumatoid arthritis (RA). Annexin a2 (Axna2) is an important mediating agent that induces angiogenesis in vascular diseases. The correlation between Axna2 and pannus formation has not been studied. Here, we provided evidence that compared to osteoarthritis (OA) patients and healthy people, the expression of Axna2 and Axna2 receptor (Axna2R) were up-regulated in patients with RA. Joint swelling, inflammation and neovascularization were increased significantly in mice with collagen-induced arthritis (CIA) that were exogenously added Axna2. Cell experiments showed that Axna2 promoted HUVEC proliferation by binding Axna2R, and could activate Hedgehog (HH) signaling and up-regulate the expression of Ihh and Gli. Besides, expression of Ihh, Patched (Ptc), Smoothened (Smo) and Gli and matrix metalloproteinase-2 (MMP-2), vascular endothelial growth factor (VEGF) and angiopoietin-2 (Ang-2), angiogenic growth factor of HH signaling downstream, were down-regulated after inhibition of expression Axna2R on HUVEC. Together, our research definitely observed that over-expression of Axna2 could promote the development of CIA, especially during the process of pannus formation for the first time. Meanwhile, Axna2 depended on combining Axna2R to activate and enlarge HH signaling and the expression of its downstream VEGF, Ang-2 and MMP-2 to promote HUVEC proliferation, and eventually caused to angiogenesis. Therefore, the role of Axna2 is instructive for understanding the development of RA, suppress the effect of Axna2 might provide a new potential measure for treatment of RA.

## Introduction

Rheumatoid arthritis (RA) is one of the most common forms of inflammatory arthritis, and it affects 0.4%-1.0% of the global population. Its characteristic pathological change is chronic synovitis. The acute early phase of RA is accompanied by the development of severe inflammatory infiltration and increased expression of pro-inflammatory cytokines, such as tumor necrosis factor-α (TNF-α) and interleukins etc., which maintain chronic inflammation of synovial tissue. The proliferation of synovial cells, synovial thickening and formation of pannus tissue, a neovascularization network of many villous projections, characterize the chronic late phase of RA. Synovial angiogenesis is the pathological basis of destruction, deformity and dysfunction of joints and is considered to be the crucial stage in the pathogenesis of RA [1]. During neovascularization, pannus formation induces invasion of cartilage and bone erosion, which represent the imbalance between anti-angiogenic and angiogenic factors in inflamed joints [2]. Many angiogenic factors are involved in angiogenesis, including MMPs, VEGF and Ang-2, which are regulated by the MAPK, PI3K and HH signaling pathways, were critical mediators among the positive regulators implicated in angiogenesis [3, 4].

Previously, our studies and those of others have showed that gene expression levels of many proteins were different in the synovial fluid of non-immune diseases such as OA, than in autoimmune diseases such as RA, including Axna2[5, 6]. Axna2, a pleiotropic calcium- and anionic phospholipid-binding protein, was one of the multigene annexin family members, and it did not belong to the transmembrane proteins. Human brain, spleen, kidney, lung and placenta tissues are rich in monomeric, heterodimeric or heterotetrameric Axna2, which were expressed on endothelial cells, smooth muscle cells, monocytes and macrophages involved in the biological processes of signal transduction, endocytosis, exocytosis and immunity globulin transportation [7,8]. In autoimmune diseases, including systemic lupus erythematous and lupus nephritis (LN), Axna2 could combine with its auto- antibodies to induce inflammatory changes [9]. Axna2 was not only involved in inflammation and immune responses, but also played an important role in metastasis and erosion of malignant tumor, especially in angiogenesis [9–13] [14,15]. Therefore, Axna2 appeared to be related to the pathogenesis of RA.

The specific pathogenesis of pannus formation in RA has not been fully identified, and an understanding of this mechanism is important in the search for a complete cure for RA. In this study, we determined that exogenously added Axna2 could promote the development of arthritis in DBA/1 mice with CIA. Further in vitro mechanistic studies showed that the Axna2/Axna2R axis promoted proliferation of vascular endothelial cells and up-regulated the expression of MMPs, VEGF, Ang-2 through the HH signaling pathway, resulting in increased angiogenesis. The results presented in this study emphasize the pro-angiogenic role of the Axna2/Axna2R axis in RA.

## Materials and Methods

### Culture of HUVEC

An immortalized HUVEC cell line was purchased from the Shanghai Cell Bank of the Chinese Academy of Sciences, and the cell lines were cultured in Dulbecco’s modified eagle medium(DMEM)(Beijing Solarbio Science &Technology Co., Ltd.) supplemented with 10% heat-inactivated fetal bovine serum (FBS) (HyClone). All of the cells were maintained at a temperature of 37°C in a humidified growth chamber under 5% CO_2_. Cell passages 1–3 of HUVEC were used to all cell experiment.

### Cell proliferation assay

The prepared cell suspension was added to 96-well plates, with 3000–4000 cells per well. The cells were stimulated with 0, 0.25, 0.5, 0.75, 1.0 and 1.5μg/ml human recombinant Axna2 (H-50, SANTA CRUZ) under 5% CO_2_ at 37°C and cultured for 24 hours. The effect of Axna2 on the cells was observed with an inverted fluorescence microscope IX71 (Olympus), followed by the addition of a cell counting kit-8(CCK-8, DOJINDO) at 10μl/well. The culture was continued for 0.5–4 hours, and we measured the absorbance values at 450nm with a 550 automatic microplate reader (Bio-Rad).

### Collagen-Induced Arthritis (CIA) in Mice

Healthy male DBA/1 (H-2q) of 7-8weeks old mice weighing 18-20g were purchased from SLRC Laboratory Animal Co., Ltd. All mice were fed in Experimental Animal Center of Second Military Medical University. Immunization-Grade Bovine Type II Collagen(CⅡ, 10mg of solution, Chondrex), Complete Freund's Adjuvant (5ml CFA, containing 4 mg/ml of M.tuberculosis, Chondrex) and Incomplete Freund's Adjuvant (5ml IFA, Chondrex) were purchased from Shanghai Bio & Mart Biological Technology Co., Ltd. The mice were divided into the following four groups: the control group (normal feeding, without injecting any agent); the Axna2 group (Axna2/CⅡ^+/-^, induced by exogenously injected Axna2 and freund); the CⅡ group (Axna2/CⅡ^-/+^, induced by exogenously injected CⅡ and freund); the Axna2+CⅡ group (Axna2/CⅡ^+/+^, induced by exogenously injected Axna2, CⅡ and freund). The dosage of the exogenously injected Axna2 depended on the dose relationship between the experimental animals and the human body surface area, each mice was injected 0.5mg/ml recombinant Axna2 50μl (25μg). The following immunization schedule was used. Induction of arthritis with a booster injection: 1) Initial injection: per mice was treated with 0.1ml emulsion mixing in equal volumes with 4 mg/ml of M. tuberculosis CFA and CⅡ (200μg) or Axna2 (25μg); 2) Booster injection: per mice was treated with 0.1ml emulsion mixing in equal volumes with IFA and CⅡ(200μg) or Axna2 (25μg) at day 21 after the initial immunization. When the booster injections, per mice injected Axna2 (25μg) in addition to injection of CⅡ(200μg) in Axna2/CⅡ^+/+^mice. Arthritis developed 4–4.5 weeks after the initial immunization, the paws of mice became swellen, and assessed the severity of arthritis by comparing the joint score, Paws thickness and the morbidity of CIA. All mice were sacrificed by cervical dislocation until arthritic mice paw swelling subsided and appeared feet deformity. All animal experiments were conducted in accordance with institutional guidelines. This study has been approved by the ethics committee (Animal Experimental Ethics Inspection of Laboratory Animal Centre Second Military Medical University) with the following numbers 13071002116. There were no unexpected deaths during the course of the experiments.

### Micro–computed tomography (micro-CT)

Radiography was performed by using the joints and paws of the DBA/1 (H-2q) mice with CIA in situ with a micro-CT scanner (Skyscan 1076; Bruker) at 35-μm resolution, with an X-ray tube current of 200 μA and a voltage of 48 kV. The 3D bone micro-architecture was generated by version 3.1 software.

### Quantitative Real-time quantitative PCR (qRT-PCR) assay

The total RNA was isolated using TRIzol (Invitrogen), according to the manufacturer’s instructions. The concentration and purity of the RNA were determined by absorbance at 260/280 nm, and the complementary DNA was synthesized using a RevertAid First Strand cDNA Synthesis Kit(K1622,Thermo). To control for variation in the messenger RNA (mRNA) concentration, the results were normalized to the GAPDH housekeeping gene. We performed qRT- PCR with the 7300 System (Applied Biosystems) and the SYBR Green Real-time PCR Master Mix (TOYOBO). The PCR amplification specificity was verified by examining the melting curve for the nonspecific peaks. We used 7300 system software to analyze the relative quantification. The primers are shown in Table 1.

**Table 1:** Primers cDNA Sequences for RT- PCR.

No.	Consensus CDS	gene	Primers cDNA Sequences
1	CCDS34153.1	Axna2R	5'-CGGAGTCTACTGGCAAAACG-3’ 5' -GCCTTCTGCTGCTATCTAAG-3'
2	CCDS6714.1	Patched	5'-CCACAGAAGCGCTCCTACA-3' 5'-CTGTAATTTCGCCCCTTCC-3'
3	CCDS5811.1	Smo	5'-GTTCTCCATCAAGAGCAACCAC-3' 5'-CGATTCTTGATCTCACAGTCAGG-3'
4	CCDS53807.1	Gli-1	5'-GGGAT GATCCCACATCCTCAGTC-3' 5'-CTGGAGCAGCCCCCCCAGT-3'
5	CCDS45487.1	MMP-2	5'-CAGCCAACTACGATGATGA-3' 5'-GTGCCAAGGTCAATGTCA-3'
6	CCDS34457.1	VEGF-A	5'-GAGGGCAGAATCATCACGAAGTGG-3' 5'-ATCGCATCAGGGGCACACAGGAT-3'
7	CCDS47761.1	Ang-2	5'-CGGCAAAATAAGCAGCATCAGCCA-3' 5'-ACCACCAGCCTCCTGTTAGCATTT-3'
8	CCDS58201.1	GAPDH	5'-GGTGGTCTCCTCTGACTTCAACA -3' 5'-GTTGCTGTAGCCAAATTCGTTGT-3'

### Gene silencing of Axna2R by plasmid transfection

The plasmid sequences for the Axna2R small interfering RNA (siRNA) were purchased from Origene (HuSHshRNA Plasmid(pGFP-V-RS) and used according to the Ronfect^TM^ DNA transfection reagent(GT2211-01, BIOMIGA) manual. After transfection for 48h, the gene expression of Axna2R was detected by qRT-PCR, and the best interference effects of the plasmid cells were passaged at 1:10 to screen for stable clones. The HUVEC screening concentration of puromycin was 1μg/ml, and the concentration was maintained at 0.5μg/ml. After three weeks, plasmid stable cell lines were established, and we used qRT-PCR techniques to identify the scrambling efficiency of the Axna2R interference group; fluorescence microscopy was used to observe the ratio of fluorescent cells. The plasmid sequences are shown in Table 2.

**Table 2:** Plasmid Sequences for Gene silencing of Annexin a2 receptor.

No.	Plasmid Sequences
TG315286A	TCTCAGACTGCCTGCCTGGAGTGGATTCTTCGC
TG315286B	CCGTGGCCTCTTCCTTTGTATCCAGTACT
TG315286C	TCACGGATCTGTGGAGCTAAGCAGCCTTA
TG315286D	GGAGTCCAGAGCACCTTGGAACCAAGTAC

### Hematoxylin-eosin(H&E) staining of slices of CIA mice joints in paraffin

We immediately removed joint tissue of the sacrificed DBA/1 mice; the tissue samples were maintained in 4% paraformaldehyde (G4011, Google Biotechnology Co., Ltd.) for 24 hours and then immersed in liquid EDTA (G1105, Google Biotechnology Co., Ltd.) for decalcification; the decalcification solution was changed once a week, until the bone tissue was easily pierced with a needle. The decalcified tissue blocks were placed in melted paraffin, in an insulated melted wax box. The samples embedded in paraffin were fully immersed in tissue blocks. The samples were cooled and hardened by solidification. Fixed the paraffin-embedded samples were fixed on a slicer and cut into a 5-8-micron thick sheet. The sheets were pasted on glass slides, following ironing by heated water, and 45°C samples were maintained in an incubator for drying later. After dewaxing and staining, we pasted the labels.

### Enzyme-linked immuneosorbent assay(ELISA) of the anti-CⅡ antibody

We collected the orbital blood of the DBA/1 mice at day 0 and at day 40 after the first immunization; the blood were centrifuged at 3,000g for 15 minutes to remove the particulates and amassed supernatant. The IgG anti-CⅡ antibody was directly measured with an ELISA kit (AMEKO, Shanghai Lianshuo Biological Technology Co.,Ltd.). Then, the different expressions of the IgG anti-CⅡantibody was determined by ELISA kits, according to the manufacturer’s protocol. A standard curve for each measurement was established using standard concentrations. Taking a blank well as zero, the absorbance was read at 450nm after adding the stop solution, within 10 min, and the result was calculated with a 550 automatic microplate reader.

### Immunohistochemistry of the paraffin-embedded CIA mice joint tissue

The immunohistochemistry was performed with an anti-rabbit/mice universal immune histochemistry kit REALTMEnVision+/HRP RABBIT / MICE (K5007, Dako Denmark A/S) manual. Each slice was covered with approximately 50μl of the CD31 antibody dilution (Wuhan Google Biotechnology Co., Ltd.), and maintained at 4°C overnight. Then, 100 μl of CD31 anti-mice antibody was added for each section and incubated for 50min at room temperature. To each slice was added 100μl of freshly prepared DAB solution, and the color was controlled under a microscope (XSP-C204, Chongqing Optical Instrument Co., Ltd.). After the color was completely gone, the samples were washed with distilled water, re-dyed with hematoxylin, differentiated with hydrochloric acid and alcohol, washed in tap water, changed back to blue with ammonia. The process continued with the slices being dehydrated (70%-100%), dried with graded alcohol, and cemented with neutral gum.

### Immunofluorescence of paraffin-embedded CIA mice joint tissue

The paraffin sections (RM2016, Shanghai Leica Instrument Co., Ltd.) were placed in a 65°C oven (DGX-9003B, Shanghai Fuma Experimental Equipment Co., Ltd.), baked for 2 h, dewaxed into water, and washed with PBS. The slices were placed in EDTA buffer to be repaired by microwave (P70D20P-TF, Galanz). We added a 3% hydrogen peroxide solution after natural cooling and incubated the solution for 10 min at room temperature, followed by 5% BSA blocking for 20 min after drying. The BSA solution was removed, and each slice was covered by diluted CD31 antibody at 4°C over-night. A mice fluorescent antibody (red 1: 100 dilution, 115-165-003; green 1:50 dilution, 115-095-003) was added, followed by incubation for 1h at room temperature. The DAPI-stained (G1012, Wuhan Google Biotechnology Co., Ltd.) nuclei were kept in the dark for 5 min; the slightly dry slices were mounted and sealed with anti-fade tablets and preserved under dark conditions at, 4°C.We used an inverted fluorescence microscope (TE2000, Nikon) for the observation and imaging.

### Statistical analysis

All statistics of experiments were analyzed by Excel or SPSS18.0 software and plotted using GraphPad Prism 5.0 or Photoshop CS 6. The data were measured as the mean ± SEM. Between the two groups were analyzed by group student *t*-test, analysis of variance was used to compare among multiple groups. For all of the tests, a *P* value of 0.05 or less was considered statistically significant.

## Results

### The expression of Axna2 and Axna2R were up-regulated in patients with active RA

**Fig 1:**
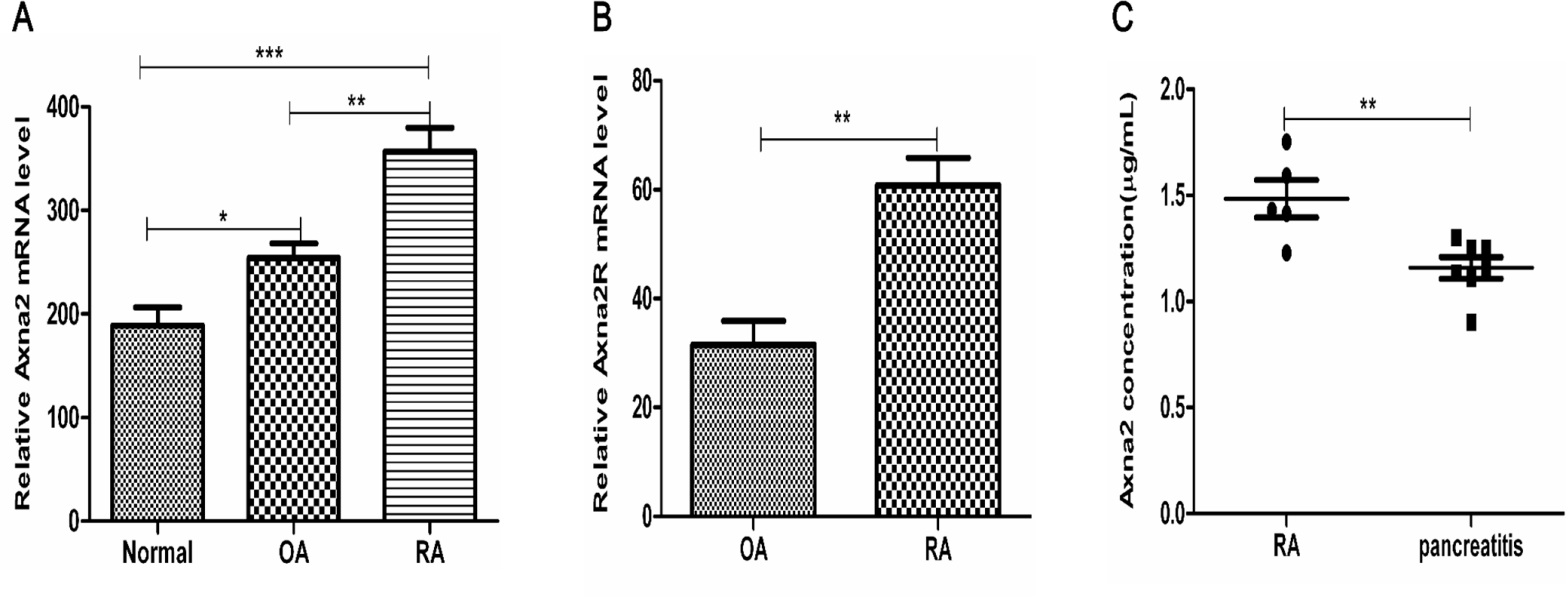
The expression of Axna2 and Axna2R were up-regulated in the synovial tissues of RA compared to OA and healthy people. **(A)** GEO DataSets showed that the expression of Axna2 mRNA in the synovial tissues of the normal controls (n = 5) and the patients with OA (n = 5) and RA (n = 5). **(B)** GEO DataSets indicated the expression of Axna2R mRNA in the synovial tissues of patients with OA (n = 5) and RA (n = 7). **(C)** The expression of Axna2 in the serum of patients with RA (n = 5) and pancreatitis(n = 7) were detected by ELISA. **(A)** *P < 0.05, **P < 0.005, ***P < 0.0005, One-way ANOVA Tukey's multiple comparisons test. **(B and C)** **P < 0.005, Mann Whitney test. The bars above showed the mean±SEM.

To investigate whether Axna2 and Axna2R gene were differentially expressed in synovial tissue between active RA and OA patients. We searched the GEO DataSets (http://www.ncbi.nlm.nih.gov/pubmed/) and found that, compared to OA, the expression of Axna2 (GSE7669, Fig 1A) and Axna2R (GSE36700, Fig 1B) were up-regulated in RA synovial tissue. These findings indicate a potential role of Axna2 and Axna2R in the pathogenesis of RA. Next, ELISA test were used to detect soluble Axna2 in serum of patients with RA and pancreatitis. The results show that the expression level of Axna2 in serum patients with RA was 1.4836±0.197671μg/ml, and in patients with pancreatitis was 1.1585±0.133205μg/ml. Axna2 were expressed higher in the serum of RA patients than that in pancreatitis, and the difference was statistically significant between the two (Fig 1C). It showed that compared to general inflammatory diseases, the expression levels of Axna2 were abnormally elevated in RA.

RA is a chronic inflammatory autoimmune disease. Axna2 has been extensively studied as a factor of inflammation initially. The relationship between cytokines and the transcripts of Axna2 showed that the expression of TNF-α, interleukin (IL)-6, and IL-1β, which are key inflammatory cytokines for maintaining the chronic inflammation in RA, was positively well-correlated with Axna2 [7] [16,17]. All of the above results implied Axna2 might play a key role in RA.

### Exogenously added Axna2 facilitated the progress of arthritis in mice with CIA

**Fig 2:**
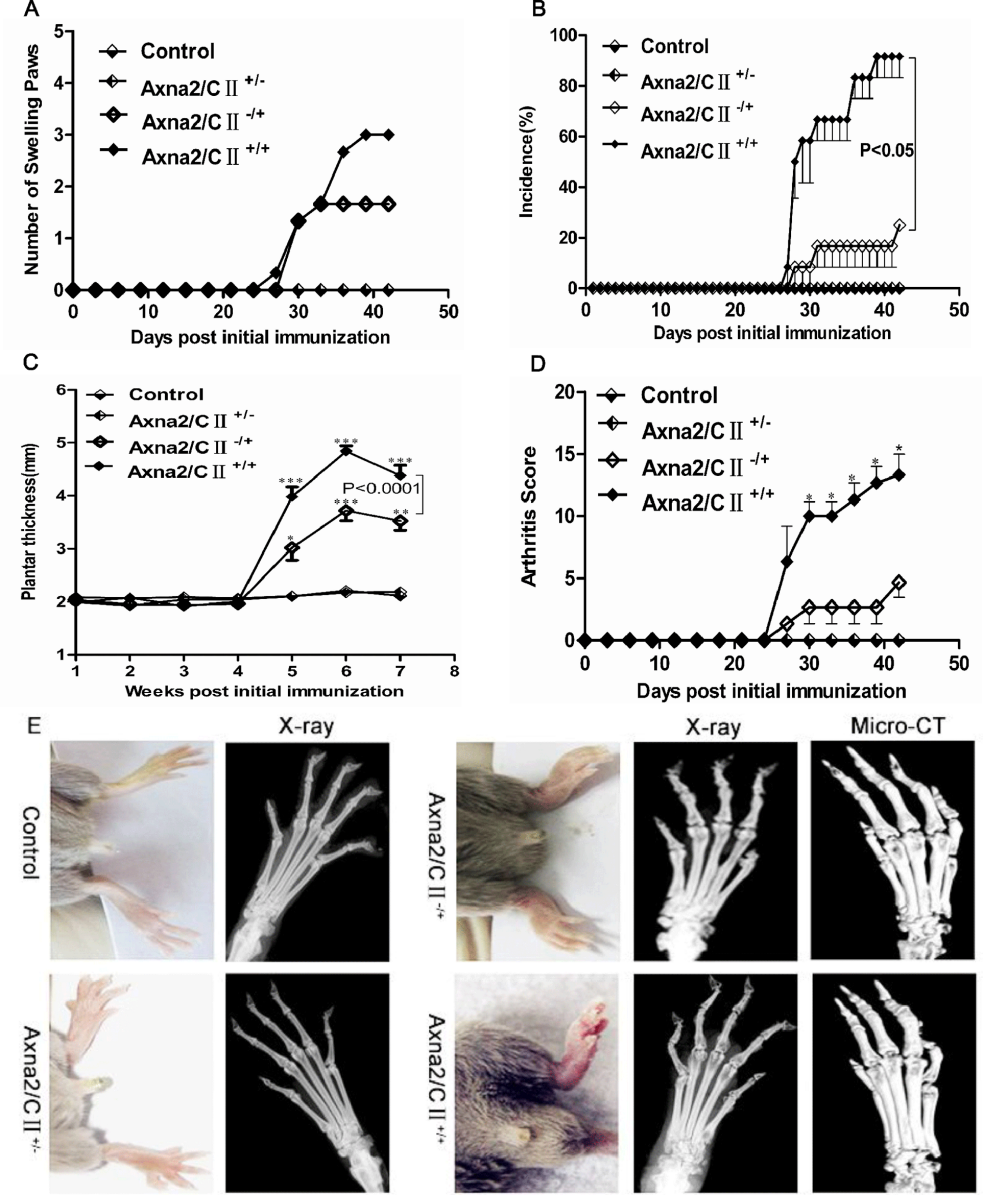
Exogenously added Axna2 facilitated the progression of arthritis in mice with CIA. **(A)** There was no swollen paws in Control and Axna2/CⅡ^+/-^ mice, the number of swollen paws in Axna2/CⅡ^+/+^ mice was larger than that in Axna2/CⅡ^-/+^ mice. **(B)** The number of swollen paws of the mice represented the incidence of arthritis. Incidence of CIA in Axna2/CⅡ^+/+^ mice was higher than that in Axna2/CⅡ^-/+^ mice. **(C and D)** Plantar thickness **(C)** and arthritis score **(D)** were clinical indicators of the arthritis changes in the four groups, the largest change happened in Axna2/CⅡ^+/+^ mice and statistically significant. **(E)** Physical photograph displayed the change of paw swelling in the four group mice. Radiography and Micro-CT of the hind paws showed the joint bone destruction of CIA mice. P value represented Axna2/CⅡ^+/+^ group compared to Axna2/CⅡ^-/+^group. **(B and D)** *P < 0.05, **P < 0.005, ***P <0.0005, Mann Whitney test. (C)**P < 0.005, One-way analysis of variance. The bars in **(B-D)** showed the mean±SEM.

To further explore the function of Axna2 in the development of RA, we caused CIA with DBA/1 mice described above. We detected whether Axna2 played an important role during the incidence and progression of RA by exogenously adding Axna2. The mice were divided into the following four groups: the Control group; the Axna2 group (Axna2/CⅡ^+/-^); the CⅡ group (Axna2/CⅡ^-/+^); the Axna2+CⅡ group (Axna2/CⅡ^+/+^). Next, we examined their clinical and histopathological features by monitoring the changes in the plantar thickness and arthritis score. The results showed that there was no incidence of arthritis without paw swelling in the Control and Axna2/CⅡ^+/-^ groups of mice. All of the mice of the Axna2/CⅡ^-/+^ and the Axna2/CⅡ^+/+^ groups presented with arthritis and paw swelling (Fig 2A). The number of swollen paws of the mice represented the incidence of CIA. However, compared to the Axna2/CⅡ^-/+^ group, the Axna2/CⅡ^+/+^ group exhibited a significantly increased incidence of arthritis (Fig 2B). Because the Control and Axna2/CⅡ^+/-^mice did not appear swollen joints, plantar thickness was not substantially changed; all of Axna2/CⅡ^-/+^ mice emerged swollen joints, but the degree of swelling was significantly lower than Axna2/CⅡ^+/+^ mice (Fig 2C). Qualitative scoring system was used to assess severity of paw inflammation. The Control and Axna2/CⅡ^+/-^ mice did not appear symptoms of arthritis, joints score was zero; swollen joints score of Axna2/CⅡ^+/+^ mice were significantly higher than that of Axna2/CⅡ^-/+^ mice, and statistically significant (Fig 2D). Next, we collected their joint tissues of being sacrificed mice to perform imaging. X-ray showed that compared to unaffected mice, the mice with arthritis appeared joint damage, bone density of the end of joint was decreased, cortical bone thinned, bone surface roughness and osteoporosis. Wherein, the degree of joint damage Axna2/CⅡ^+/+^ mice was more serious than Axna2/CⅡ^-/+^ mice. 3D scans of the bone micro-architecture with computed tomography (Micro-CT) revealed that compared to Axna2/CⅡ^-/+^ mice, the fragmentation index, structure separation were increased and percent porosity were reduced in Axna2/CⅡ^+/+^mice, which showed the more severe of articular bone destruction of Axna2/CⅡ^+/+^ mice (Fig 2E). These observations demonstrated that Axna2 was not an initiation factor of the incidence of arthritis; however, exogenously added Axna2 could promote the development of arthritis, including aggravated arthritis and bone destruction. It indicated that Axna2 might play an important role in the development of RA.

### Exogenously added Axna2 enhanced the inflammatory changes and promoted angiogenesis in mice with CIA

**Fig 3:**
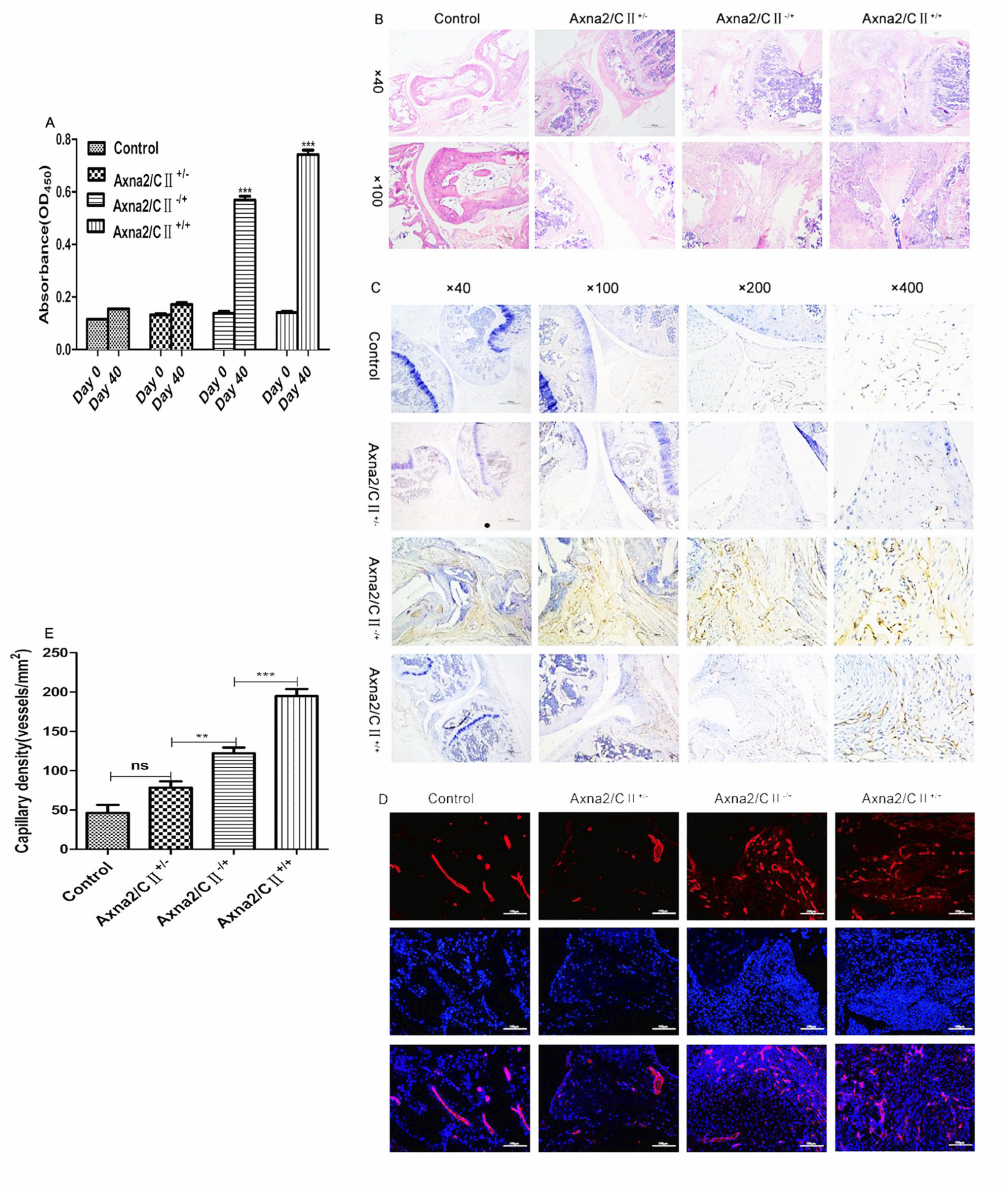
Axna2 enhanced the inflammatory changes and promoted angiogenesis in mice with CIA. **(A)** The differential expression of the CⅡ-specific antibodies between the early initial immunization (day 0) and the peak of arthritis (day 40) in the serum of the CIA mice. **(B)** H&E staining of joint tissue showed a remarkable increase in synovial inflammation as well as cartilage and bone destruction in the Axna2/CⅡ^+/+^ mice compared to that in the other three groups(original magnification×40,×100 with an electron microscope). **(C and D)** The IHC **(C)** and IF **(D)** (original magnification×200) analysis of the CD31 by using fluorescence microscopy. The number of neovascularization was larger significantly in Axna2/CⅡ^+/+^ mice than the other three groups. **(E)** Three vies of the original magnification×200 were selected for the quantitative analysis of the expression of CD31. The highest expression of CD31 was found in the Axna2/CⅡ^+/+^ group. *P*<0.05 represented a significant statistical difference. **(A)** ***P < 0.0001, Two-way ANOVA, experiments were repeated at least 3 times. **(E)** **P < 0.005, ***P < 0.0005, One-way analysis of variance. **(A and E)** the bars in showed the mean±SEM.

Immune and inflammatory responses have been described as essential in the pathogenesis of CIA. CⅡ-specific antibodies have been suggested to correlate positive well with the severity of CIA [18]. Therefore, we collected orbital blood at day 0 and 40 after the initial immunization. ELISA was used to analyze the total CⅡ-specific antibodies in serum of CIA. The results showed that compared with unaffected mice, the serum levels of CⅡ-specific antibodies were significantly elevated in mice with CIA at day 40 after the first immunization, especially a highest expression of CⅡ-specific antibodies in the serum of the Axna2/CⅡ^+/+^ group and statistically significant (Fig 3A). It indicated indirectly that Axna2 might enhance the severity of CIA.

To determine which aspect of the progression of arthritis is affected by Axna2, we collected samples of the joint tissues of CIA mice and used H&E staining for the analysis. The histopathological analysis showed that compared with the other groups, inflammatory cells in joint synovial tissues of Axna2/CⅡ^+/+^ mice were increased, the edge of articular bone was not sharp, indicating a more severe inflammatory changes and cartilage and bone destruction in the Axna2/CⅡ^+/+^ mice (Fig 3B).

Because neovascularization could damage the bone structures of joints, the purpose of this research was to clarify the relationship between Axna2 and pannus formation of RA. Therefore, we performed immunohistochemistry (IHC) and immunofluorescence (IF) to analysis the formation of neovascular in joint tissues of CIA mice depending on observing the expression of surface marker of microvascular endothelial cell—CD31. The expression of CD31 represented the capillary density in the synovial tissue of CIA. The results showed that there were very little neovascularization exist in the joint tissue of the Control and Axna2/CⅡ^+/-^ groups, as well as much less than that in the Axna2/CⅡ^-/+^ and Axna2/CⅡ ^+/+^ groups. In particular, in the Axna2/CⅡ^+/+^ group, the amount of new microvessels was significantly increased over that in the Axna2/CⅡ^-/+^ group (Fig 3C and 3D). In order to reflect the results of above experiments directly, we selected three images of 200 resolution to perform immunohistochemical quantitative analysis of CD31 in each group, and found that angiogenesis of joint tissue in Axna2/CⅡ^+/+^ mice were much more than in Axna2/CⅡ^-/+^ mice, and significant difference(Fig 3E). It further validated that Axna2 might promote angiogenesis in mice with CIA.

### Axna2 combined with Axna2R promoted proliferation of HUVEC

**Fig 4:**
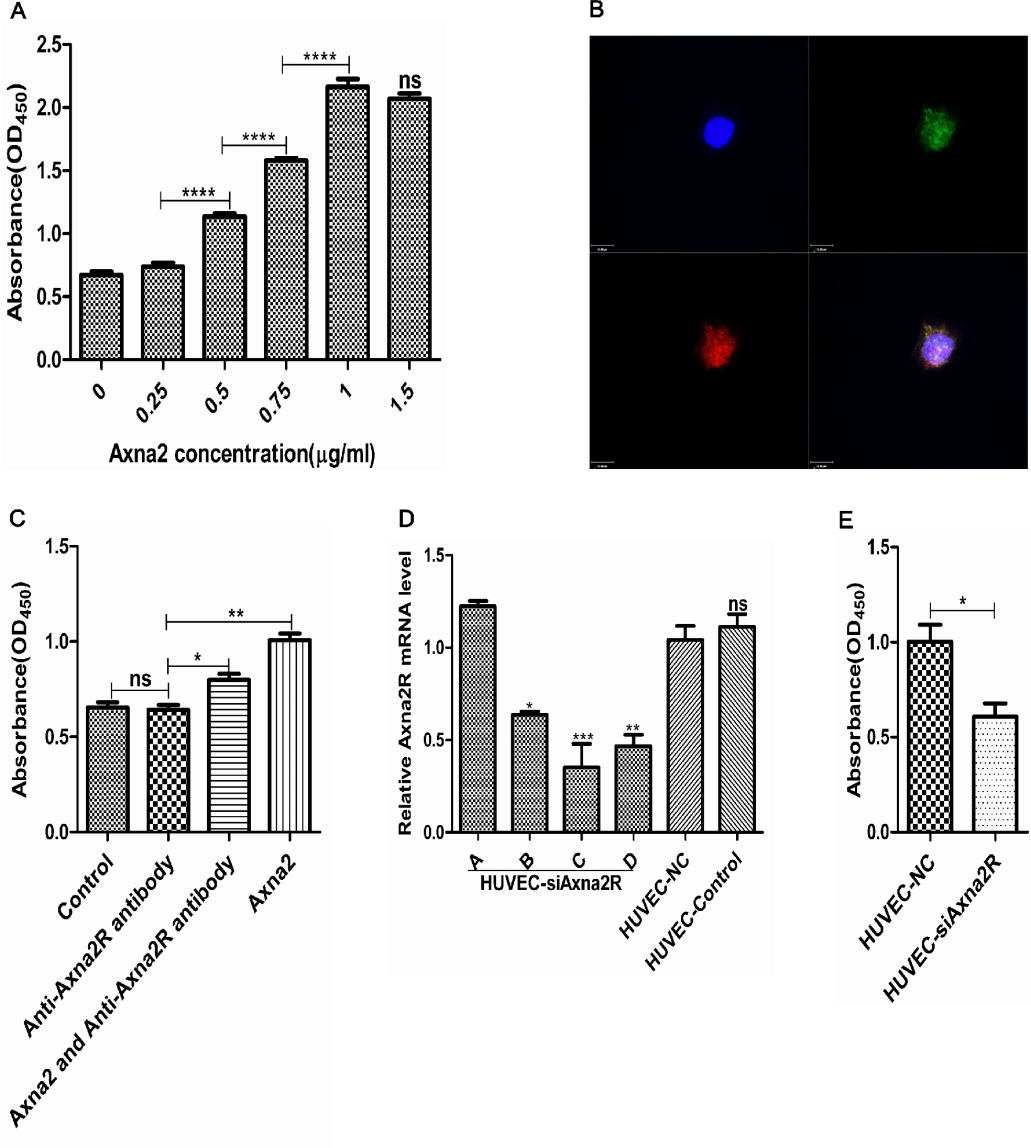
The relationship between Axna2/Axna2R and proliferation of HUVEC. **(A)** Axna2 concentration-dependently promoted HUVEC proliferation, and at concentration of 1.0μg/ml achieved the maximum effect. **(B)** HUVEC cells were incubated with an anti-Axna2 antibody (green) and an anti-Axna2R antibody (red). Axna2 was marked by GFP, and Axna2R was marked by RFP. The staining was visualized with confocal microscopy. **(C)** After blocking Axna2R with the anti-Axna2R antibody, the promoting proliferation of Axna2 were weakened partly. **(D)** Detected the effect of plasmid transfection of knocking-down expression of Axna2R by using RT-PCR technology. plasmid C was selected for the following experiment based on the results of this experiment.**(E)** After silencing of Axna2R by plasmid C, the effect of the proliferation of Axna2 weakened.**(A, C and D)** *P < 0.05, **P < 0.005, ***P < 0.0005, One-way ANOVA Tukey's multiple comparisons test. **(E)** *P < 0.05, Mann Whitney test. Experiments were repeated at least 3 times. The bars above showed the mean±SEM.

Pannus formation was pathological basis of joint bone destruction, and neovascularization is the main characteristic change for pannus in RA. To investigate the specific mechanism of over-expressed Axna2 and Axna2R during pannus formation in RA, we co-cultured HUVEC with different concentrations of Axna2 in vitro. CCK-8 proliferation assays were used to detect the absorbance values at OD_450_. The results showed that the OD_450_ absorbance values of HUVEC were positively correlated with the concentration of Axna2 and gradually up-regulated along with the increasing concentrations of Axna2, achieved maximum effect at 1.0μg/ml (Fig 4A). It suggested that over-expressed Axna2 could promote the proliferation of HUVEC.

Because Axna2 not belongs to the transmembrane proteins, we aimed to determine whether Axna2 promoted proliferation of HUVEC depends on Axna2R that located on the surface of target cell membrane. We first performed confocal laser scanning for HUVEC. The images showed that Axna2R was predominantly located on the cell membrane (Fig 4B). Herein, we added 1.0μg/ml of the anti-Axna2R antibody into cultured HUVEC to close the effects of Axna2R on the cell membrane and compared the OD_450_ value of CCK-8. The results showed that there were no significant statistically difference in the degree of cell proliferation between experimental group and control group. It demonstrated that the anti-Axna2R antibody had no apparent toxic effects on HUVEC, suppressed Axna2R alone did not affect the proliferation of HUVEC. Next, we incubated HUVEC with the anti-Axna2R antibody to end the effect of Axna2R on the cell surface of HUVEC at first, and then added 1.0μg/ml Axna2 to stimulate HUVEC for 24 hours. We found that the anti-Axna2R antibodies could partly weaken the Axna2 effects on cell proliferation with a significant statistical difference by CCK-8 (Fig 4C).

To investigate the specific signaling pathways in the Axna2/Axna2R axis, we used plasmid transfection technology to knockdown the expression of Axna2R on the surface of HUVEC (HUVEC-siAxna2R) (Fig 4D). Next, we used the same concentration of Axna2 (1.0μg/ml) to stimulate the HUVEC-NC group and the HUVEC-siAxna2R group of plasmid C. The results showed an increasing difference of extent of the cell proliferation in the HUVEC-NC group and statistically significant (Fig 4E). These results demonstrated that extracellular Axna2 could concentration–dependently promote the proliferation of vascular endothelial cells, and this effect partly contributed to Axna2 combine with Axna2R on the surface of endothelial cell membrane.

### The Axna2/Axna2R axis might benefit angiogenesis through HH signaling

**Fig 5:**
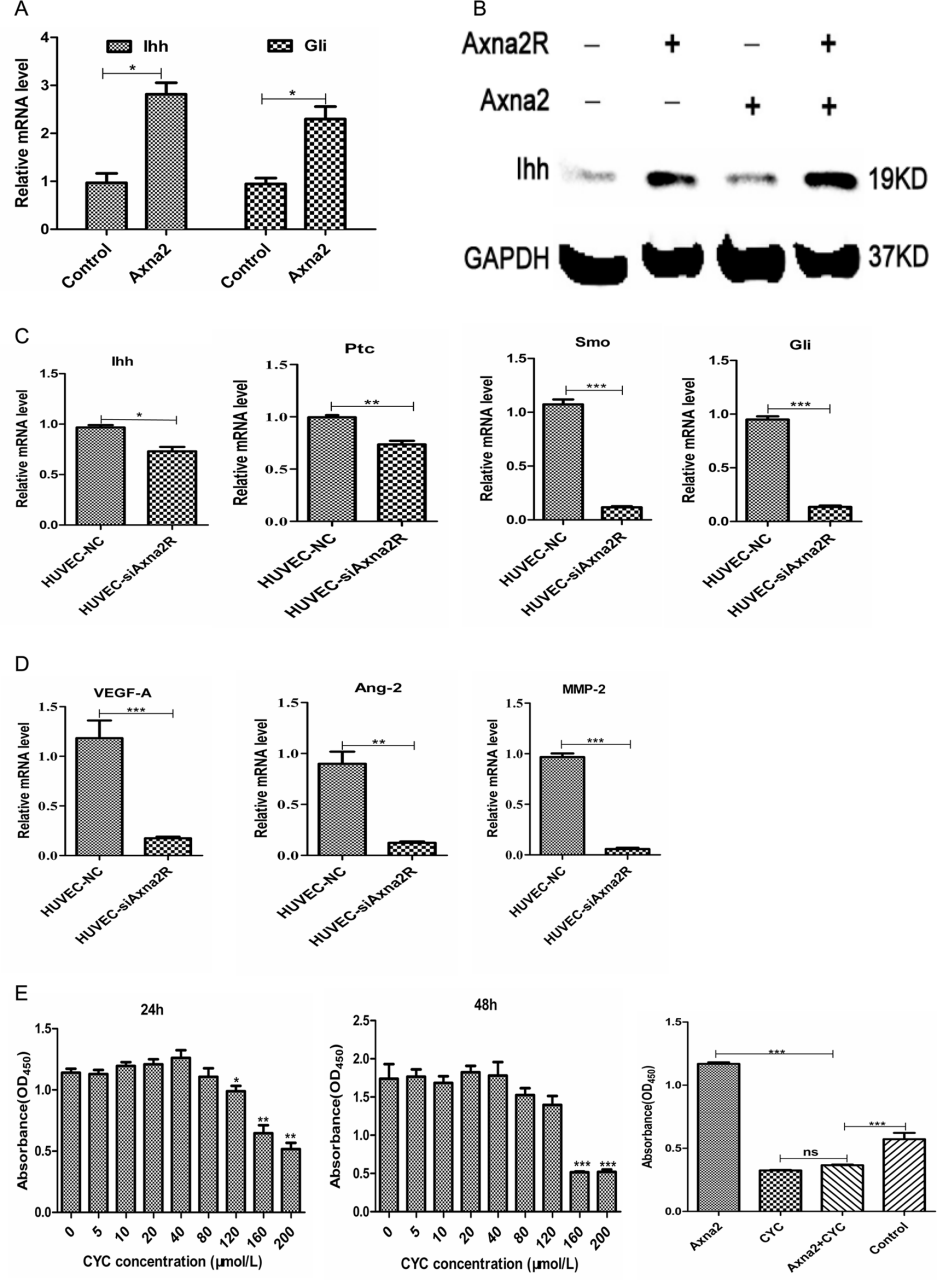
The Axna2/Axna2R axis affected HH signaling. **(A and B)** The expression of Ihh and Gli increased after being treated with 1.0μg/ml Axna2 for 24 hours by using RT-PCR and western blotting. **(C)** Knockdown of Axna2R on the surface of HUVEC could down-regulate the expression of Ihh, Ptc, Smo and Gli. **(D)** Knockdown of Axna2R on the surface of HUVEC decreased the expression of HH signaling downstream MMP-2, VEGF and Ang-2. **(E)** After inhibition of HH signaling by CYC, the effect of Axna2 in promoting endothelial cell proliferation was significantly decreased. The bars showed the mean±SEM. **(A, C and D)** *P < 0.05, **P < 0.005,*** P < 0.0005, Mann Whitney test. **(E)** *P < 0.05, **P < 0.005,*** P < 0.0005, One-way ANOVA Tukey's multiple comparisons test. Experiments were repeated at least 3times.

In order to clarify the specific signaling molecules mechanism of Axna2/Axna2R axis in promoting proliferation of HUVEC. We reviewed a number of relevant literatures and found HH signaling could be activated in RA synovial tissue, and activated HH signaling benefited the proliferation of synovial cells [11]. Therefore, we first treated HUVEC with 1.0μg/ml Axna2 to observe the differential expression of junction molecules of HH signaling. The results showed that the mRNA expression of Ihh and Gli, the key molecule HH signaling, were increased in HUVEC cells that treated with Axna2 (Fig 5A). It indicated that Axna2 could activate HH signaling pathway and induced the expression of downstream Gli. The intriguing point is significantly higher levels of Gli in protein level for 24 hours (Fig 5B), but Gli no charge in protein level at this time.

Next, we used plasmid C to suppress Axna2R of HUVEC and detected HH signaling. Compared to the HUVEC-NC group, the mRNA expression of Ihh, Ptc, Smo and Gli, the critical node molecules of HH signaling, was significantly reduced in the HUVEC-siAxna2R group (Fig 5C). At the same time, the mRNA expression of MMP-2, VEGF and Ang-2, which were considered to be crucial pro-angiogenic factors, the downstream molecules of HH signaling were decreased in the HUVEC-NC group than in the HUVEC-siAxna2R group (Fig 5D). Besides, we co-cultured HUVEC cells with the specific inhibitor of HH signaling—cyclopamine (CYC) at different concentrations for 24 hours and 48 hours. Experiments of CCK-8 were used to analyze the relationship between HH signaling and proliferation of HUVEC cells. The results showed that the OD_450_ values decreasing with the increasing of CYC concentration, indicating diminishing proliferation of HUVEC caused by CYC, but there was no difference between 24h and 48h group (Fig 5E). It suggested that CYC could inhibit the proliferation of HUVEC. On this basis, we started stimulate HUVEC with CYC for 24h, and then added 1.0μg/ml Axna2 continue co-culture for 24h. We found that Axna2 promotion of endothelial cell proliferation was significantly weakened after the inhibition of HH signaling (Fig 5E). These results suggested that extracellular Axna2 could activate HH signaling pathway of HUVEC; Axna2/Axna2R axis regulated the expression of the key nodes molecule of HH signaling Ihh, Ptc, Smo and Gli, thereby regulated the expression of pro-angiogenic factors MMP-2, VEGF and Ang-2 in HUVEC cells, and led to endothelial cell proliferation finally.

## Discussion

Previous studies have reported that Axna2 play a critical role as paracrine or autocrine factors in autoimmune diseases [19]. In antiphospholipid syndrome, β2GPI bound Axna2 as high affinity sites on endothelial cells to crosslink with aPL/anti-β2GPI antibodies, triggering the intracellular signaling pathway and inducing inflammation or thrombosis [20,21].Yung S *et al*. found that the anti-dsDNA antibody bond to mesangial cells depends on Axna2 entering the cytoplasm and nucleus, activating the P38-mitogen-activated protein kinase (MAPK), c-Jun amino-terminal kinase (JNK), and AKT pathways, which induced IL-6 secretion and Axna2 synthesis in a destructive cycle, promoting the development of lupus nephritis [13]. The latest study reported that Axna2 was a critical mediator in discoid domain receptor-2/ Axna2/MMP activation, which might promote proliferation of the synovial fibroblasts as well as articular damage [22].

The role of Axna2 in neovascularization has been described, particularly in tumor diseases. Axna2 was involved in the pathogenesis of hepatocellular carcinoma, gastric cancer, lung cancer, breast cancer, and pancreatic cancer as well as in the prognosis of advanced cancer [23–28]. During the pathogenesis of these malignancies, the over-expression of Axna2 acted as a co-receptor of plasminogen and the tissue plasminogen activator, which could convert plasminogen to plasmin and contribute to increase plasmin activity on the tumor cell surface, mediate extracellular matrix degradation and promote neoangiogenesis, resulting in the promotion of tumor growth [29–31]. Semov A *et al*. reported that Axna2 binding and co-localization with S100A10, mediated S100A10 to promote tumor invasion, metastasis and angiogenesis [32]. Zhou S *et al*. suggested that up-regulated expression of Axna2 promotes angiogenesis by the HIF-1α/VEGF-A pathway [33]. To treat neovascular- related diseases, several individuals have attempted to cause the drugs to bind Axna2 to inhibit the role of Axna2 in angiogenesis [34].

Neovascularization is initiated by pro-angiogenic mediators that promote the release of proteolytic enzymes including matrix metalloproteinases, the process results in degradation of the endothelial cell basement membrane and the outer perivascular extracellular matrix. Angiogenesis is a hallmark of RA, and pannus formation is one of the key elements of the invasion of cartilage and of bone destruction. Persistent angiogenesis leads to chronic changes in the architecture of the RA synovium, affecting the delivery of nutrients and inflammatory cells and regulating the production of cytokines and protease activity. Previous research has shown that endocan, the CC chemokine system, tumor necrosis factor-α, and connective tissue growth factor as well as the angiogenesis-related molecules, cadherin-11 and HIF-1α, are involved in pannus formation in RA. Phosphorylation of Akt and p38 MAPK, nitric oxide and the HH signaling pathway, particularly NF-κB, are important regulators of inflammation-induced angiogenesis. However, the understanding of the role of Axna2 in neovascularization in RA remains limited. To identity the relationship of Axna2 with angiogenesis in RA, we established CIA model with DBA/1 mice. CIA shares both immunological and pathological features with human RA, therefore it has been used extensively as a model to study the pathogenesis of RA and for testing therapeutics [35]. The results of mice experiments showed that exogenously added Axna2 could contribute to the progression of arthritis. IHC and IF analysis of CD31 supported the finding that Axna2 significantly enhanced angiogenesis in mice with CIA.

To further study the pro-angiogenic function of Axna2 *in vitro*. Different concentrations of Axna2 were co-culture with HUVEC. The results indicated that over-expressed Axna2 could promote proliferation of HUVEC by binding to Axna2R. Although research has shown that the Axna2R could induce apoptosis independent of Axna2 in the cytoplasm, the only known function of Axna2R is to bind to and regulate the Axna2 signal on the cell membrane [36,37]. It has been demonstrated that the Axna2/Axna2R axis promotes the adhesion, growth, invasion and metastasis of cancer cells through the MAPK, ERK1/2 and AKT pathways [38].

During the progression of RA, angiogenesis was critical in maintaining tumor-like synovial growth and an inflammatory state. Inhibition of angiogenesis is a major therapeutic target for RA [39–41]. Several mechanisms might regulate pannus formation by different signaling pathways or angiogenic factors in RA. Among the known positive regulators of angiogenesis, VEGF and Ang-2 are critical mediators of normal and abnormal angiogenesis. Several investigations have reported that the concentration of VEGF in synovial fluid is significantly higher in patients with RA than in patients with OA or other forms of arthritis. Genetos DC *et al*. indicated that VEGF regulated the expression of Axna2 by the Src and MEK kinase pathways in the absence of oxygen [13].

Many studies have shown that HH is involved in tumor invasion, metastasis and angiogenesis [42–44]. The delivery of HH signaling depended on two receptors on the target cellular membrane, Ptc and Smo, and activated the nuclear transcription factor Gli. In the inactive state, the Ptc protein bound to Smo, which belonged to the transmembrane protein, and inhibited the activity of Smo. Once the HH ligands binding to Ptc, HH signaling pathways is activated, facilitates the release of Smo and prevents the function of Ptc, then activates the translation of the target molecules by the waiting translation factor of the Gli family members.

It has been reported that HH signaling pathway could regulate normal cell growth and differentiation, which is required for tissue homeostasis and regeneration in adults. While perturbed HH signaling is associated with human cancers, promoting cell proliferation, motility, invasion and angiogenesis, and it could be a potential therapeutic target in malignancies [43][45,46]. Besides, HH signaling were activated in RA, and promoted fibroblast-like synoviocytes (FLS) proliferation of synovial tissues [47]. It is likely to play an important role in the angiogenesis of RA. Our research clearly identified that Axna2 could activate and magnify HH signaling pathway and induced increasing expression of downstream Gli and Ihh; the expression of Ihh, Ptc, Smo and Gli, the key nodes of HH, as well as the downstream pro-angiogenic expression of VEGF, Ang-2 and MMPs were significantly reduced after being knock-downed expression of Axna2R of HUVEC. Therefore, Axna2/ Axna2R axis might be linked to Hedgehog activation and affected angiogenesis.

The Axna2 tetramer, which was released by osteoclast-like cells, mediated the functions of macrophages depending on TLR-4 [48]. In bone marrow, it acted as an autocrine/paracrine clastogenic factor on bone cells and activated human macrophages by induction of MAPK and nuclear factor (NF)-κB signaling and the production of pro-inflammatory cytokines [23]. This finding entirely confirmed that Axna2 was positively correlated with TNF-α, IL-1β and IL-6, which were the key inflammatory cytokines that maintain the inflammatory state in RA [11]. The ultimate result was the destruction of bone in RA. The over-expression of Axna2 increased the alkaline phosphatase activity, facilitating the osteoblastic mineralization process. The pro-inflammation factors, TNF-α and IL-1β, enhanced the mineralization process, which were dependent on the JNK of the MAPK signaling pathway [49–51]. However, Axna2 also could up-regulate the expression of granulocyte monocyte-macrophage colony-stimulating factor (GM-CSF) and the NF-κB-activating factor receptor ligand (RANKL) in human bone marrow, which were important to promote formation of osteoclast (OCL), and led to bone destruction by undermining the balance of osteoblasts (OBL) and OCL [52]. These findings appeared to be contradictory, and an investigation of the roles of OBL and OCL in the specific pathogenesis of bone destruction in RA is needed.

**Fig 6:**
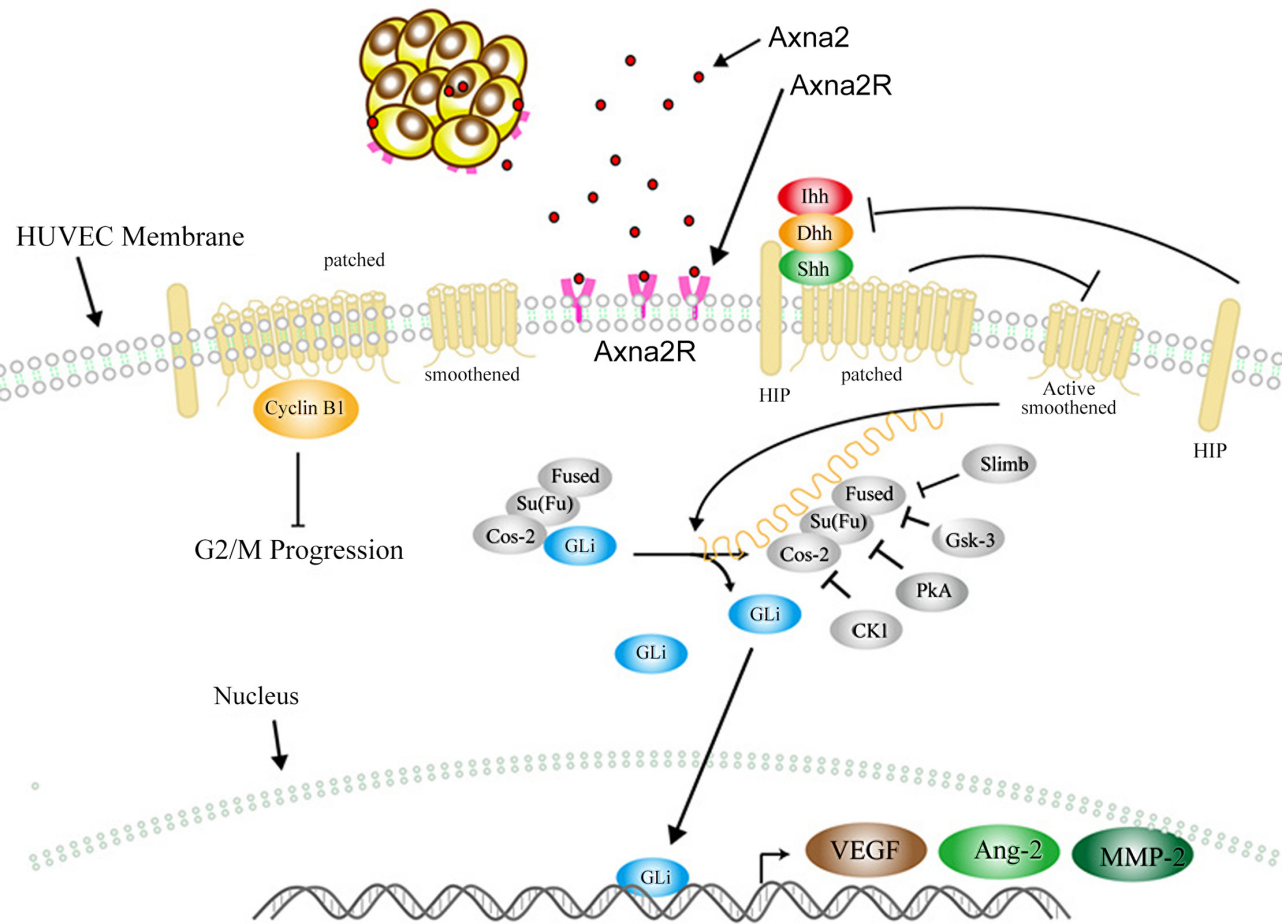
Function of Axna2/Axna2R axis in promoting neovascularization of RA. In the pathogenesis of RA, the over-expression of Axna2 on vascular endothelial cells could bind Axna2R and activate the HH signaling, causing increasd expression of angiogenic factor, and ultimately promote neovascularization.

Our study demonstrated that Axna2 could promote the progression of RA. Axna2 played an important role in pannus formation of RA. The cytological analysis showed that the Axna2/Axna2R axis significantly promoted neovascularization by activation of the HH signaling pathway and increased the Ptc, Smo, and Gli expression to up-regulate the expression of the downstream MMPs, VEGF and Ang-2. These results suggest that the effect of Axna2 might provide a new potential measure for treatment of RA (Fig 6).
